# Induced myelopathy after long-term anti-tumor necrosis factor alpha treatment

**DOI:** 10.1186/1546-0096-9-S1-P111

**Published:** 2011-09-14

**Authors:** C Modesto, F Caracsegui, S Melendo, A Sanchez-Montanez, Manel Roig

**Affiliations:** 1 Department of Pediatric Rheumatology, Hospital Vall d’Hebron, Barcelona, Spain; 2 Pediatric Emergency Unit, Hospital Vall d’Hebron, Barcelona, Spain; 3 Department of Pediatric Radiology, Hospital Vall d’Hebron, Barcelona, Spain; 4 Department of Pediatric Neurology, Hospital Vall d’Hebron, Barcelona, Spain

## Background

Treatment with anti-TNF (tumor necrosis factor) agents has been effective in many inflammatory conditions. However, severe adverse events can occur, even after years of therapy. Damage of central nervous system has been well documented in adults.

## Aim

To draw attention to the possibility of neurological complications of anti-TNF therapy in children.

## Methods

Clinical and radiological discussion of a 13 yr. old boy who presented to our Emergency Unit due to the impossibility for standing up or walking in the last 24 hours. During the previous days he had noticed some paresthesias in the lower extremities and difficulty initiating urination and defecation. Physical examination revealed normal muscle tone and tendon reflexes in his upper extremities, but severely diminished muscle strength in the lower limbs, hyperreflexia and exhaustible clonus. Cranial and spinal cord MRI performed the same day revealed the existence of two lesions, one at the cervical level (C2-C4) and another at the medullary cone (T12-L1), showing enhancement after contrast media administration. Diagnosis of demyelinating disease was performed. Treatment with etanercept had been started eight years before together with methotrexate due to the existence of inflammatory polyarthritis (Blau syndrome).

## Results

Etanercept was discontinued and the patient treated with high-dose intravenous corticosteroids during five days, followed by oral prednisone for six weeks. Functional recovery was achieved after four weeks.

## Conclusion

Central nervous system damage related with TNF blockade could appear also in children, even in those patients who did not present any side effect for prolonged periods of time.

